# Book Reviews

**Published:** 1819-11

**Authors:** 


### CRITICAL ANALYSIS

OF

### RECENT PUBLICATIONS, IN THE DIFFERENT BRANCHES OF MEDICINE AND SURGERY;

### SELECT MEMOIRS, AND HISTORIES OF CASES;

### In the Literature of Foreign Nations.

Πατρίδες ἄρα
Ἀνδράσιν, οὐ πατραῖς ἄνδρες, ἀγαλλόμεθα.

---

***Hints of a new Idea of the Nature of the Cellular Tissue.*** **By G. M. De Felici, m.d. Public Demonstrator and Conservator of the Collection in the Museum for Pathology and Comparative Anatomy, in the University of Padua. 8vo. Padua, 1817.***

ALTHOUGH, as we have already observed, it was our intention to give a generally comprehensive view of the pathological doctrine at present prevalent in Italy,† before we particularly noticed treatises on distinct subjects; yet we have been induced to deviate from that intention on the present occasion, from the observations contained in this little pamphlet being almost the only ones of importance relating to the structure of the elementary tissues, that have been advanced since the time of Bichat; and from the consideration that they may be useful to the reader in his reflections on the work of Dr. Baron. We shall present our readers with the substance of it, without adducing any particular observations of our own, excepting some references to authors who have treated on, or produced illustrations of, the same subject.

The cellular texture enters into the formation of every other part of the human body; and of many of them it constitutes the greater proportion of their structure. It was long since considered by some physiologists as a net-work of vessels, more or less pervious to the fluids; but this opinion has not been generally adopted. Haller regarded it in a different manner: he says, "*Vasa, quæ tunicas pingunt, accessio sunt cellulosæ telæ, neque constituunt naturam membranæ, sed membranæ per cellulosam telam innatæ superadduntur.—Cellulosa fabrica fibris fit laminisque solidis omnibus, neque cavis, neque vasculosis, etsi vasculis adcedentibus pingatur.*"‡ A similar notion was entertained respecting it by Bohn,§ Bordeu,|| and

---

* *Cenni di una Nuova Idea sulla Natura del Tessuto Cellulare.* Del Dottore G. M. De Felici, Pubblica Rèpertitore è Custode de' Gabinetti Patologico e di Notomia Comparata, nella I. R. Universita di Pavia. 8vo. Pavia: Bizzoni. MDCCCXVII.

† We are waiting for some important works that have appeared since our observations were made and arranged; and this will occasion the postponement of our Essays on this subject to the ensuing volume of this Journal.

‡ *Primæ Lineæ Physiol.* c. i.

§ *De Continuationibus Membranorum.* Amstelodamiæ, 1763.

|| *Recherches sur le Tissu Muqueux, ou l'Organe Cellulaire.* Paris, 1767.

BICHAT,* the principal of the original authors on this subject who have written since Haller. But Dr. Felici observes, the very accurate observations of some great anatomists seem to show the correctness of the first-mentioned opinion; and he adduces some peculiar to himself, that contribute to establish its propriety.

"RUISCH," the author says, "had already observed, that the interior parenchymatous structure of the spleen was nothing else than an aggregate of vessels; and he showed some specimens of that organ, injected according to his own mode, in which nothing could be seen but an admirable tissue of vessels, ramified in a prodigious manner; as SABATIER† has also stated. HEISTER injected, by the veins of the penis, the glans and corpora cavernosa, which were thus rendered in appearance a plexus of venous vessels.‡ Similar injections made by Prof. REZIA are preserved in the anatomical cabinet in the university of Padua. But the conclusion I shall hereafter advance, is also founded on some other observations made by Prof. CAV. MORESCHI.

"The corpora cavernosa of the penis, from the most careful and repeated observations and injections made in the human subjects, and various animals, especially in the horse of Prof. PANIZZA, appear to be nothing more than a mass of vessels, forming layers, anastomosing with each other, and which give to those parts the spongy, alveolar, or cellular, appearance, those bodies present. The other cavernous structures, that of the clitoris as well as that of the papillæ of the breasts, are obviously of the same nature. The cavernous tissue which surrounds the vagina, *plexus vaginam pene totam ambiens*, is merely formed by a multitude of vessels, transmitted from the adjacent parts, communicating with each other by frequent and short anastomoses. The cavities seen within the texture of the uterus communicating together, which much resemble those of the spleen and the corpora cavernosa of the penis, and which are enlarged and distended during the time of menstruation and pregnancy, are also shown to be only vessels passing into that organ in a serpentine direction. The truth of these observations is clearly demonstrated by the beautiful preparations in the Anatomical Museum in our University."

May we not then consider, says Dr. Felici, that the cellular tissue is formed in the same manner as the spongy texture of the spleen, and the corpora cavernosa of the penis? Are not, making an abstraction with respect to size, the net-works, the cellules, and the alveoli, the same in one as in the other? And why should we then regard their formation in a different manner? And are there not many circumstances which show the correctness of the opinion above advanced?

"If we observe the back of the hand," the author continues, "we see a tissue constituted solely of an extremely fine vascular net-work. All the arteries which are distributed in the membranes have also the same reticular figure. '*Fines arteriarum*,' says Haller, '*cylindrici, aut cylindricis proximi, ramulos producunt in eadem longitudine crebriores, plerumque in rete dispositos, ut quilibet ramus minoribus*

---

* *Anatomie générale. Système Cellulaire.* Art. i. § 1.

† *Traité complet. d'Anatomie,* tome iii. *de la Rate.*

‡ *Compendium Anat.* tab. v. fig. 22, 25.

*surculis cum utroque vicinorum anastomoses faciat. Ita in omnibus membranis reperitur.*' And, speaking of the veins, he adds, '*Rami majores venarum magis reticulati sunt, ac frequentioribus anastomosibus, nec inter parva, sed inter maxima vasa, inter vicinas, dextras et sinistras, superiores et inferiores venas, ubique adparentibus coeunt.—In minoribus ramis, retibusque membranosis, viscerumque interiori fabrica arteriæ, venæque plerumque conjunctæ ambulaut. Minus fere flexuosæ sunt.**

Many beautiful and fine injected preparations in the Museum at the University at Padua, some visible to the naked eye, others only by the aid of the microscope, as well as others furnished by Professor Scarpa, show in the most evident manner the cellular tissue of the face, the palms of the hands, the soles of the feet, the villi of the intestines, &c. to be nothing else than a congeries of vessels. "How surprising are the injections with mercury of the lacteals of the small intestines of the turtle, in the specimen contained in our Museum. In this preparation, presented by Prof. Scarpa, the lacteal vessels are so numerous, that it might be said the intestines of this animal are merely a congeries of those vessels; the metal shining through every discernible point of their surface. These injections would frequently succeed if the necessity of injecting the lymphatics by the branches, leaving the trunks to avoid the opposition of the valves, did not present a forcible obstacle.

"The injections of Ruisch prove the same thing; and yet these injections, however happily they may have succeeded, never fill the whole of the most minute vessels."

The beautiful observations of Prof. Scarpa, too, on the nature and generation of the bones, prove that they are not formed by lamellæ, or fibres, as formerly believed, but by a reticulated, tomentous, cellular tissue:† these, with the injections of the bones in general made by the same physiologist,‡ seem to show that the basis of the osseous structure is nothing but an aggregate of vessels.

These notions respecting the formation of the cellular tissue are powerfully supported by the phenomena attending the formation of new parts in the healing of wounds, the union of fractures of the bones, the generation of false membranes and cysts, which are apparently wholly formed by fine vessels shooting into the effused lymph. These are circumstances that at least show the subject to be worthy of attention, in the manner the author has here pointed out. Dr. Felici considers that it is from an alteration in the state of these vessels, that thin, diaphanous, and pellucid, membranes become dense and opaque in inflammation. "Thus," he says, "the fine and transparent lamina of the conjunctiva, by dilatation, varicosity, elongation, and obstruction, of the veins, becomes thick and opaque, pulpy, flocculent, and of a reddish hue; so that, on a first view, it would appear that a new membrane was generated on the cornea."

Whenever the cornea is affected with cloudy specks, a congeries of

---

* *Primæ Lineæ Physiologiæ*, c. ii. §§ 38, 48.

† *De Penitiori Ossium Structura Commentarius*. 4to. Lipsiæ, 1799.

‡ See *Index Rerum Musei Anatomici Ticinienis Antonii Scarpa*.

varicose venous vessels will be found on the white part of the eye, corresponding to those specks more elevated and convoluted than the other sanguineous vessels of the same order; and, if the cornea be clouded in more than one point of its circumference, there will be seen so many distinct plexuses of varicous venous vessels distributed on the eye, exactly corresponding with them. It appears, on a first view, that each of those venous congeries, so distinct from, and prominent above, all the rest, have forced a passage for the blood through the boundaries of the sclerotica to the cornea. "I have an eye preserved," says Prof. Scarpa, "taken from the body of a man affected with chronic varicose ophthalmia, accompanied with cloudiness of the cornea, who died of inflammation in the chest. Having injected the head by the arteries and by the veins, I found that the wax, with which the veins of the conjunctiva were well filled, had found a free passage, as well in the more elevated congeries of those veins, as in the more minute branches of the same congeries running in a serpentine direction on the surface of the cornea, in the precise spot where the cloudiness had existed; whilst, in all the rest of the circumference of the cornea, the injected wax was arrested by having found, in the confines of the cornea and the sclerotica, an insuperable obstacle. And it is wonderful to see in this eye, by the aid of the lens, the extremely fine net-work formed by the numerous venous branches in the limits between the cornea and the sclerotica, where they beautifully anastomise together in a thousand ways all around it, without any of them, excepting in the spot corresponding to the situation of the opacity of the cornea, passing the boundaries formed by strong adhesion, which the fine lamina of the conjunctiva there receives. The *pterygium* is originally produced by an increased dilatation of minute venous vessels passing over a particular part of the surface of the cornea; to this is then added a greater thickness than natural, and an opacity of the fine lamina of the conjunctiva which covers the cornea, on which those vessels are spread. The facility and promptness of the cure in these cases, by excision of the congeries of vessels on the conjunctiva, and a concentric incision at the margin of the cornea, by which this regains its original pellucidness in the short space of twenty hours, is a proof that this disease is kept up more by a varicose and elongated state of those vessels, still pervious, than by an effusion of serum or coagulable lymph in the delicate lamina of the conjunctiva which is spread over the external surface of the cornea."*

On this principle,—that is, from the generation of new vessels, or from the elongation of the extremities of those of the inflamed part,—the author observes, it is that the generation of new membranes in inflammation depends; and he refers to preparations in the Museum of the University of Padua, in which membranes of that kind, formed in peripneumonia, carditis, pericarditis, and abdominal inflammation, were filled with mercury by injection. Preparations illustrating this vascular union in the morbid adhesions occurring in inflammation,

* See the new edition of the *Saggio di Osservazioni e di Esperienze sulle Principali Malattie degli Occhi,* di Antonio Scarpa, capi 7, 8, 11.

have been presented to it by Scarpa, Borda, and Joseph Frank. The following case contributes to the support of these opinions.

On dissecting a body, accompanied by Prof. Panizza, Dr. Felici says that he found a large, isolated, cystoid, tumor, in the left hemisphere of the brain, which contained about eight ounces of coagulable lymph mingled with pus of yellowish-green colour. The lateral ventricle, which was adjacent to it, was driven considerably out of its natural situation, in a downward and backward direction. The cyst which contained the fluid above mentioned, could not, apparently, have been formed by the membranes of the brain. On examining it particularly, Prof. Panizza found it to be composed of two laminæ, extremely vascular: the external one adhered to the cerebral mass by ramifications of vessels; the other presented a granulated appearance on its internal surface, was of a red colour, and looked indeed as if it had been injected.

"These facts (the author continues) will explain the nature of the tubercles found in the skin, which are merely a net-work of vessels, as is demonstrated by injections, with the exception of some nervous fibrili; which, though we cannot trace them, we know to exist from their effects.*

"We may thus explain the generation of new parts in wounds, by the elongation of the divided extremities of vessels into granulations, which restore the soft parts to their original form: the same means also produce the union of bones.

"We may thus explain the obstinacy of ophthalmia, the opacity and condensation of the conjunctiva in chronic varicose ophthalmia, in cloudy specks on the cornea, and in pterygium, by the excessive distension suffered by the vessels of the conjunctiva during the inflammatory state; by the varicosity and elongation of the vessels, principally the venous; and, consequently, by the condensation of the conjunctiva produced by the inflammatory action.

"We may thus explain the generation of new membranes, the morbid adhesions between them and other parts, and the origin of cystoid tumors within the substance of the brain.

"We may thus also account for the *vasa vasorum*, which enter into the formation of the cellular tissue of the vessels themselves, of which Haller observed, '*Arterias arteriæ habent in extremo imprimis celluloso textu, a vicinis undique arteriosis trunculis natas, multas, ramosas, reticulatas, omnes exiguas, in fetu etiam absque injectione plurimas.*'†

"The same facts will enable us also to comprehend more satisfactorily the sudden redness that tinges the face in the act of blushing, as well as the paleness of the skin and the cutis anserinis from terror, or from cold; by the influence of the nervous action on the contractile power of the arteries, and the varieties in the transmission of the blood by them which thence arise.

---

* Caldani, *Institut. Physiol.* c. 16, §§ 224, 225, 227. Fattori, *Guida alla Studio dell' Anatomia Umana*, §§ 367, 368.

† *Prim. Lin. Phys.* c. ii. § 32.

"We may thus explain the growth, the preservation, and the death, of animals, according as these vessels are elongated, and give way to the impulse of the blood, or by degrees successively become closed and obliterated; and, lastly, according as their excitability shall become destroyed by the influence of stimulants.

"Lastly, we may thus more easily explain the secretions over the whole external and internal surfaces, and in the cavities of the body, and their re-absorption; and the readiness with which adipose matter is deposited, as Haller observed, '*Via ab arteriis ita proxima et libera est in cellulas adiposas, ut majuscula ostia eo hiare necesse sit, quæ dimittant mercurium, aerem, aquam, gelatinam, oleum, semper etiam in vivo animale pigrum. Non per longos aliquos propriæ fabricæ ductus excernitur, sed per totam arteriæ longitu dinem undique exsudat, ut nulla pars circumpositæ cellulosæ telæ absque madore sit, quando arteria aqua repletur. Adeps calidus in arterias pulsus easdem vias facillime legit. Celeriter congeritur, argumento cito renascentis post acutos morbos obesitatis.*'*

"Considering, then," the author concludes, "the whole of this extensive tissue, not as a texture of no importance, except as a basis for sustaining the figure and firmness of the body, but as an aggregate of vessels, the immediate organ of the secretions, of nutrition, of absorption, &c.; and viewing it entering into the formation of, and furnishing life to, every part of the system; we shall form a more noble, and probably more correct, idea of its uses in the animal economy."

These notions, Dr. Felici is careful to observe, are not advanced in a confident manner, but with the intention of exciting the attention of anatomists to some new researches on the subject. The observations he has adduced, seem to be sufficiently precise and important to lead to such an investigation; and the views they disclose of the structure of the body generally, certainly must facilitate our reasonings on what we witness of its functions.

---

***Discourses on the Elements of Therapeutics and Materia Medica.*** By NICHOLAS CHAPMAN, M.D. Professor of the Institutes and Practice of Physic and Clinical Practice in the University of Pennsylvania, &c. 2 vols. 8vo. 1817 and 1819.

[*Continued from p.* 166.]

IT will not be necessary for us to extend our observations on the question of the absorption of medicinal substances from the skin and the intestinal canal, in order to show that this is effected more commonly than Dr. Chapman has supposed. It may be proper, however, to remark, that, in our arguments against his doctrine, we employed only those facts that had been known and published before the period when these discourses were composed: the discoveries that have since then been made respecting absorption by the venous system, will probably be alone sufficient to lead Dr. Chapman to alter his opinion on this subject. The question, however, how far the effects

---

* *Prim. Lineæ Phys.* c. i. § 19.

ordinarily witnessed from certain medicines depend on their diffusion through the system, or on sympathetic connexion, remains open for investigation; but many new researches are requisite to furnish the means for satisfactory conclusions. We now commence with the discourse on *the classification of the materia medica;* but a desire to pass on to that part of the work devoted to practical illustrations, will render our remarks on this subject but very concise: we shall confine them to a few general reflections, chiefly relating to the classification adopted by the author.

Dr. Chapman very properly separates the consideration of articles of diet from that of medicines; not that the qualities of the former are unworthy the attention of the physician, but because they should form a distinct subject for investigation.

After some remarks on the difficulties attendant on a classification of the materia medica, from the same article being expressly emetic, cathartic, diaphoretic, &c. under different circumstances, he notices the errors of that of Cullen; which is, indeed, quite inconsistent with his own doctrine of *solidism.*

Dr. Chapman then exposes that which he has himself adopted, which, as he acknowledged in his Preface, is not free from important errors, and is by no means the best that could be formed in the present state of medical knowledge. He, in the first instance, states that every medical substance is more or less stimulant to the animal economy. This proposition must be admitted, generally considered; but it is not a good foundation for the classification of them which Dr. Chapman has adopted,—that is, into *general* and *diffusible* stimulants. A few observations will render this evident. Wine applied to the stomach under certain states of disease, and even of relative health, will lower the temperature of the skin and render it pallid: here, then, it acts locally as a stimulant, but remotely as a sedative; a phenomenon arising from sympathetic connexion and concentration of the vital powers. On the other hand, cold air applied to the skin will, on some occasions, increase the powers and actions of the stomach, while it renders the skin pallid, from the causes already mentioned. A blister, too, is frequently the most powerful sedative that can be employed, from its withdrawing the vital actions from a remote part by the local irritation it produces. And, finally, certain substances applied to the stomach will increase the actions of remote parts in one state of the body, and depress them in another; phenomena depending chiefly on the greater or less plethoric state of the system, and the different degrees of vitality with which it is, under various circumstances, endowed.

There is another objection to this method, that has not escaped the penetration of Dr. Chapman; which is, that even the local agency of certain medicinal substances that is stimulant in the state of health, will be, apparently, relatively sedative in certain states of disease. He observes, "as whatever produces any positive impression on the living system, must do it by an irritant power, there can be no doubt that, in a very strict sense, every medicinal substance is a stimulant. But the effect thus created may be inferior to the natural degree of excitement, and much less than that of disease. When this happens, as lessening action, the article may with propriety be denominated, and in a practical view considered, sedative, in contradistinction to our more energetic remedies."

Their mode of agency may be thus regarded: the substances in question, by producing the actions dependant on their own specific influence, supersede the morbid actions, or the actions to which every part is excited by the influence of the rest of the economy.

This principle is practically applied to a great extent by many of the most eminent Italian physicians of the present day; and they administer tartar emetic, several other of the metallic salts, opium, &c. to parts suffering morbid action, even of an inflammatory kind, from the views thence derived.

Reasoning on it is not very satisfactory, but actual observation seems to show its truth. The eye, under certain forms of inflammation, will furnish a ready means for the illustration of this subject. This doctrine of *counter-stimulation* was first advanced by Rasori, on renouncing *Brownism*. Considering inflammation as the sign of increased action, and yet being obliged to acknowledge that topical stimulants sometimes remove it, it became necessary to resort to the reasoning we have above alluded to.

According to the present state of our knowledge, the most correct and useful classification of the materia medica must be that proposed by Bichat; which Dr. Chapman acknowledges by saying, "I am now persuaded that a much more natural, as well as useful, arrangement of medicines might be made, on the principle of their affinities to the several systems of the body; and, should an opportunity be ever afforded me, it is the one which I shall attempt to establish."

M. Alibert, whose fame is secured by the records of so many branches of medical science, has done much for the development of those views; but it will require the labours of successive generations to render them complete.

Dr. Chapman has in some degree modified his plan on those principles; for, though he divides medicinal substances into the two classes of *local* and *diffusible* stimulants, he treats of those coming under the former according to their influence on particular functions,—as emetics, cathartics, diuretics, lithontriptics, diaphoretics, expectorants, emenagogues, anthelminthics, and epispastics. In a perfect system of this kind, it would be necessary to comprise the particular influence of medicines on the elementary textures of the body; for it seems evident that certain ones affect particularly the serous, others the mucous membranes, &c. The second class of Dr. Chapman includes tonics and astringents. We shall first enter into the consideration of the former.

Previously to his observations on the use of *emetics*, the author adduces some remarks on the act of vomiting, and on the way in which this is excited by certain substances. Physiologists now seem to agree in the opinion, that, concurrent actions of the stomach itself, the diaphragm, and some of the abdominal muscles, ordinarily take place in this act. Perhaps the difficulties the author notices in accounting for the agency of emetics, may be obviated by referring this to that law of the animal economy, by which every organ enters into a sort of action calculated to remove what is injurious to it; and, by motions tending towards the surfaces of the body, rather than such as may transmit them further into the system, when the matter is of a noxious quality. Thus, we find that substances irritating to the stomach, and not fit for the exercise of its digestive functions, are ejected from it by vomiting, if deleterious to the general economy, and transmitted to the bowels, if not so; whilst alimentary matter is pertinaciously retained until it is properly elaborated. These seem to be general laws, although they are often apparently diverged from; probably, in consequence of the common outrages committed against nature by civilized man in respect to his food.*

Emetics, Dr. Chapman observes, influence the system in various ways besides that depending on the mere removal of the contents of the stomach. They incite the liver and gall-bladder, secretion and evacuation of bile, and probably the pancreas, in a similar manner. From the sympathy of the stomach with the rest of the system, various morbid actions are averted by the powerful action and subsequent repose of this organ. The skin is peculiarly affected on this occasion, and is generally incited to increased secretion of a salutary tendency. The pulmonary organs are also called into action in a similar manner. The powerful effects of emetics in promoting absorption have been long observed, although not satisfactorily explained. The rational doubts that have lately been advanced respecting the influence of the lymphatic vessels on these occasions, especially as relates to the removing of collections of purulent matter, &c. must turn our attention to what seems to be the true means, but of which we have not yet sufficient knowledge to enable us to form any conclusions.

"In fever," Dr. Chapman says, "when the attack is exceedingly vehement, whatever may be the type, we should never fail to resort to an emetic, and even to repeat it, if a very successful impression be not made on the case by the first exhibition. This precept, however," he adds, "is more applicable to the bilious fevers of our own climate, and especially as they occur in the southern States, where they prove exceedingly intractable under any other mode of treatment." This observation is particularly interesting, as coming from a physician who considers that fever, "whatever may be the cause, is always a disease of sympathy, having the primary link of its ultimately lengthened and complex chain in the stomach. It is upon this organ that contagion, marsh effluvia, and other noxious matters, act; and hence, precisely as in the cases of poison, a local irritation at first occurs, which, if not at once arrested, spreads itself by multiplying the trains of morbid association, till the disease becomes general, involving more or less every part of the animal economy." Those who have adopted the doctrine of the gastric origin of several species of fever, have in

* See the fortieth volume of this Journal, pages 531, 534, 535, 536, for some interesting illustrations of this subject.

general been much afraid of the use of emetics, considering that they are hazardous remedies at any stage of the disease of the species alluded to; and Dr. Chapman, indeed, very strongly urges the importance of restricting the administration of them in what are called *malignant fevers*, to "the forming state:" it is in this way only, he says, that they are admissible. "Exhibited in the more advanced stage of the case, or after the disease is absolutely confirmed and pervades the system, they not only prove wholly incompetent to its removal, but generally heighten the worst symptoms, and augment the difficulty of cure. This effect was most remarkably exemplified in the treatment of our yellow-fever, and has been remarked in some other epidemics,—as the plague, &c." Whilst we state our dissent from the author's universal* application of a correct and important view of the origin of fever, as regards the seat of the "primary link of the morbid chain," we must express our conviction of the truth and value of his remarks respecting the use of the remedy under consideration. We believe it is solely from its having been employed after real inflammation of the gastric passage has come on, that it has fallen into its present disrepute. Reasoning, as well as experience, leads us to conclude, that an emetic will very frequently subvert the morbid action before it has acquired this degree of intensity; but, after this is established, emetics are commonly injurious: this distinction should have been more clearly made.

In puerperal fever, Dr. Chapman has prescribed emetics with advantage, "not so much to meet the general indications of the disease, as to relieve one most distressing symptom. The stomach is here not unfrequently loaded with a dark offensive matter, resembling the black vomit of our pestilential fevers, which occasions great distress and sickness, and, if allowed to remain, uniformly keeps up fever, and depresses the system into a typhoid state. To evacuate this noxious matter, an emetic is indispensable; and its operation, in some instances, will be followed by effects the most prompt and satisfactory." His opinion respecting the nature of this disease, is, that it has its origin in a primary irritation of the uterus; and that the peritoneum, in the more violent cases, takes on inflammation from sympathy.

The difference between the state of the uterus resulting from the nervous irritation causing this malady and real inflammation, is well pointed out by the judicious and experienced Giannini; and his views of this subject have led him to adopt a mode of practice that appears to have been extraordinarily successful. The remedy on which he has placed most reliance, is the cold, or the *semi-tepid*, bath, according to the state of the patient, the season of the year, and the stage of the disease. His expression relative to its utility is very strong: he says, "when the disease is treated by cold immersion, all other remedies may be dispensed with as useless, in the greater number

* From other parts of this work, we are led to suppose this to be an inadvertent expression; and that the author means only to apply it to what are called malignant fevers, with the consideration of which the paragraph in which this expression occurs commences. Dr. Chapman's remarks on the nature of Puerperal Fever seem to prove this.

of cases." The auxiliaries he resorted to were slight tonics, laxatives, and opium.*

Emetics have not preserved the repute they once acquired in the cure of hæmorrhage from the lungs; and Dr. Chapman joins in the opinion that their use on those occasions is hazardous: though, he says, the worst case of this kind he ever witnessed was completely suspended by a dose of digitalis that produced violent vomiting. In the more slight and chronic forms of this affection, they may be exhibited with safety, and are often very beneficial.

Dr. Chapman makes a distinction between menorrhagia and menstruation, however profuse: in the former, he considers the discharge to be really blood; whilst that occurring in the latter, is the result of a secretory action of the uterus. He has never employed emetics in uterine hæmorrhages, but very frequently nauseating doses of the medicine, and with a complete confirmation, in his mind, of what had been previously said respecting the efficacy of this measure. Dr. Chapman refers particularly to the statement of Bergius, and Althof of Upsal; and states that he has witnessed several cases, where, "the moment the nausea was induced, the flooding ceased." He prefers ipecacuanha for this purpose, and frequently combined it with opium, in the quantity of two grains of the former with half a grain of the latter, giving this dose every two or three hours. The same medicine was employed in dysentery with equal benefit, confirming the statements of Cullen, Sir George Baker, Mr. Playfair, Mr. English, Mr. Hamilton of Ipswich, and Mr. Joseph Arnold.

In croup, the author thinks very highly of their use, more so than we believe is generally done. There is often great difficulty in exciting vomiting in severe and sudden attacks, especially when it is of a spasmodic, rather than an inflammatory nature. On this occasion, Dr. Chapman says, "My experience teaches me that nothing is so effectual as the warm bath; and, where it fails, venæsection, in extreme cases, even *ad deliquium animi.* Never have I witnessed one solitary instance in which these means combined did not succeed in awakening the susceptibility of the stomach to the action of the emetic, and effecting all which can be expected from the most free and copious vomiting."

In the malignant sore-throat, and in complaints of the chest in general, the author concurs in the common opinion in favour of their considerable utility.

In rheumatism, he has not had much experience of their use, excepting in that species "originating in districts exposed to marsh exhalation, and where the attack, as sometimes happens, is blended with intermittent or remittent fever. They here prove serviceable, on a principle perfectly intelligible." In dyspepsia, his "first step is generally to resort to this remedy." We have ourselves seen much

* See *Della Natura delle Febbri, e del Miglior Metodo di Curarle,* &c. Del Dottore Guiseppe Giannini, Medico nello Spedal Maggiore di Milano; tom. ii. p. 117. We shall take another opportunity of noticing this physician's observations and opinions on this and several other febrile diseases, in a more particular manner.

mischief produced by it, especially where the patients have been accustomed to too free a use of spirituous liquors and irritating food; and these are such common causes of dyspepsia, that we think some restriction is requisite in the use of emetics. The application of leeches and vesicatories to the epigastric region, and a very restricted diet, are, in by far the greater proportion of cases, more efficacious, as well as more safe, remedies than tonics.

In what are called nervous affections, the author considers this medicine as among the most important of our means in their management. He considers "that it is highly probable, that every one of the series has its origin very often in the alimentary canal, and sometimes in the stomach itself." M. Broussais, by his pathological researches before and after death, has left this no longer a matter of doubt.

"Of the class of neuroses," says Dr. Chapman, "the one which appears to me sometimes to be most unequivocally a gastric affection, is epilepsy. Entertaining this impression of the nature of the disease, I have freely prescribed emetics in it, and with manifest advantage. By exhibiting them just before the accession of the paroxysm, they will often prevent it; and even if they do not altogether resist it, they render it milder and of shorter duration. Nor is this all which they accomplish. By the strong and direct impression made on the stomach, the commencement in that organ of the wrong association constituting the disease, is broken; and afterwards the case yields to those remedies which we denominate tonics." This view of the subject will again be noticed when we arrive at the discourse on purgatives.

We pass over some less important observations on the use of emetics in apoplexy, palsy, and sick head-ach, to those relating to mania.

Dr. Chapman first notices the difficulty with which vomiting is here excited in many instances; and he remarks that its removal, as in croup, is a pretty certain sign of convalescence. The fact is, it is a sign that the balance of vital action is more accurately restored. To meet this indication by a succession of the most active and stimulating emetics, as the author advises, is, we think, a hazardous measure: those of the mineral species should, at least, not be chosen for the purpose; for, although the relations of the stomach to the brain, by means of which vomiting is excited, may be much disordered, what Bichât termed the *organic sensibility* of that organ may exist in its full force, and serious mischief will arise from this being put into action by strong stimulants, without our being advised of it by pain; and from the very same cause that vomiting is not excited. Of this we have witnessed several instances. The vegetable emetics should here be chosen if this remedy be thought advisable.

Of the efficacy of emetics in what the physicians of the United States termed *mania à temulentia*, of which we gave some remarkable instances a short time since in a memoir by Dr. Klapp, the author cannot speak from personal observations, "having always been so well satisfied with a different plan of managing the disease, which I shall hereafter mention when I come to discuss the properties of opium, that I have not been induced to deviate from it in a single instance."

Hydrocephalus internus, Dr. Chapman says, he is convinced from attentive observation of its phenomena, as well as from the lights of dissection, does not, most commonly, depend on an accumulation of water in the ventricles of the brain, "but consists in an altered state of that organ, arising from a primary derangement in the chylopoietic viscera, and particularly of the stomach."

On this subject, as on almost every other, the author's opinions, when not original, are conformable to those of the best modern pathologists. We, however, regret that he has not oftener referred to their sources those whom he has borrowed; but this is an impropriety expressly dependant on the publishing these discourses in their original form of Lectures. We shall recur to this subject hereafter. The author seems to be more inclined to the use of emetics in this disease than Dr. Cheyne, and those who have adopted his opinions. One of his views in their use, is, however, peculiar to himself: he says, "I should administer them on a very different principle from that on which they have heretofore been supposed to operate. It would be to make an impression on the parts in which I presume the disease to be seated, and not to promote the absorption of effused fluids." They have not been commonly used for this purpose, but rather to remove offending matter from the stomach. Dr. Chapman's notion may furnish a useful indication in the early stages of the disease.

In the severe painful affections of the exterior integuments of the cranium, usually termed cephalalgia, Dr. Chapman has employed emetics with benefit. "Early adopting the notion that this complaint proceeds from a morbid condition of the stomach, the only two cases of it which have come under my care I managed by emetics, and had reason to be entirely pleased with the result. Even genuine tic douloureux, the neuralgia of some writers, has been cured, in several instances, by this same practice, and with such facility as to place it decidedly above all the modes of treating this most painful, and hitherto untractable, affection. The credit of employing emetics in this case seems due to Dr. Physick."

Richter, Dr. Chapman observes, maintained that many diseases of the eyes, especially amaurosis, proceed, more or less, from a disordered state of the chylopoietic viscera, chiefly the stomach. No one of the time had half his reputation in the management of that complaint, and, of course, not equal experience. His fame, indeed, was so diffused, owing to his unrivalled success, that persons afflicted with it resorted to him from all the countries of Europe. He deduced his practice directly from the theory of the disease which he had adopted. Considering it primarily a gastric affection, he directed the free exhibition of emetics, and afterwards a combination of tartarized antimony, with some other articles, to keep up a constant impression on the stomach.

The more attentively we examine the extant histories of this affection, the climates in which it is most prevalent, and the class of persons who are particularly subject to it, the more fully we shall be convinced of the correctness of those views.

In connexion with these observations respecting the origin of amaurosis, we should remark, that M. DEMOURS, the most eminent oculist in France at this time, has witnessed some cases where it came on during the existence of diabetes, and, as he considers, from the same causes as those which produced the latter affection.

The following observations are particularly interesting. Dr. Chapman says, "Depraved vision I have several times seen from spasms of the stomach; and one case, perfectly well authenticated, is in my possession, where total blindness took place, and continued for many hours, in consequence of a severe attack of bilious colic, and which was ultimately removed by copious evacuations from the alimentary canal."

On the use of emetics in the different varieties of dropsy, the author's remarks are less important. He refers to Richter for a case of diabetes which succeeded to fever, that was cured by this remedy.

"Emetics have been prescribed in the bites of venomous animals, inducing constitutional affections. This was originally an Oriental practice; but I have understood that it is at present a good deal followed throughout our western country, where people are often bitten by the rattle-snake and other poisonous serpents. Of the utility of the remedy I cannot speak from my own knowledge; though, as in all cases of morbid poisons, the stomach is always very seriously, if not principally, affected, I am disposed to entertain very favourable impressions of the remedy."

Emetics are very frequently employed by the Chinese physicians, and we have somewhat to learn from them, we are inclined to think, respecting the attention to the state of the stomach in many diseases. This is a subject we shall formally enter into the consideration of at no very distant period.

With a reference to the opinions respecting the efficacy of emetics in strangulated hernia, and in favouring the reduction of dislocations, and the following remarks on their use in difficult parturition, "dependant on rigidity of the passages," as it is here expressed, the author concludes this discourse: "I have tried this means, and have seen the experiment made by others. The effect was, a very great degree of general relaxation and distress, without at all facilitating the dilatation of the parts, or in any manner promoting the labour; and hence the practice has been abandoned."

We have indulged in the foregoing extensive transcriptions from this part of the work, because the observations of the author are throughout judicious, and, when not original, so important, that they can hardly be too frequently impressed on the mind of the practitioner; and because the use of this remedy seems to have been much, and we think unjustly, neglected of late years on this side of the Atlantic. We earnestly advise, at least the younger part of our readers, to peruse attentively Dr. Chapman's remarks on this subject. Our limits will not permit us to extend our extracts respecting it much further: these must now be made from the discourse on the *particular emetics.*

Amongst the author's remarks on the particular application of different species of emetics to different cases, it may be useful to adduce the following: When mere evacuation of the contents of the stomach is desired, ipecacuanha is the most appropriate, as it generally comes up with the first vomiting, leaving the stomach in a state of composure, if tepid fluids are not afterwards drunk; and, for this reason, it may also be given in a very large dose without inconvenience. Tartar emetic, on the contrary, generally produces repeated vomiting, and acts, moreover, on many other functions; and, for these reasons, is most applicable to cases where the production of the sympathetic effects of vomiting is also required.

We have, however, been taught to adopt greater reserve in the use of the latter in children, than Dr. Chapman inculcates, particularly in cases where vomiting is excited with difficulty; having seen very serious injury arise from a large quantity of the medicine being retained in the stomach. It is a convenient, but often a dangerous, emetic for exhibition to young children.

Cullen, who contributed so much to introduce the tartar emetic again into use, maintained, as Dr. Chapman observes, that it produces no advantage unless it vomits, or creates considerable nausea. Fordyce, however, remarked, that it was most efficacious in febrile disorders, when it produced the slightest gastric disorder; and in this opinion Dr. Chapman concurs; and he very judiciously remarks, that "medicines seem to do good in the cure of fever, by exciting their own specific or peculiar action; and, when they disorder the stomach by sickness, they depart from this, and, if they do not act as poisons, always become nugatory, or more or less mischievous." During the continuance of nausea, arterial action, muscular power, and animal temperature, are undoubtedly lowered; though, the moment it ceases, there is uniformly, at least in febrile affections, a re-action of the system, and a correspondent exacerbation of the disease.

We have abundant and forcible proofs of the great efficacy of tartar emetic in severe diseases, without any evident effect on the stomach being produced, in the useful observations of Dr. Balfour of Edinburgh, on this subject.*

Locatelli was accustomed to depend almost solely on the use of this medicine in the cases of typhous fever admitted into the hospital at Milan, and with the most favourable results. We have much to learn from the Italians respecting the use of this remedy in various inflammatory diseases, and in many of those vaguely termed *nervous;* but we shall shortly notice in a particular manner the works of Prof. Tommasini, and this will lead us to the consideration of this subject.

Sir George Baker and Sir John Pringle, the author remarks, preferred the use of antimony to ipecacuanha, in the treatment of dysentery; but he himself is disposed to prefer the latter. Of their use in diseases of the skin, he speaks in very favourable terms; agreeing, however, with Dr. Willan, that they must be employed for a considerable length of time to be productive of much permanent benefit.

---

* *Observations, with Cases, illustrative of the Sedative and Febrifuge Powers of Emetic Tartar.* By Wm. Balfour, m.d. Edin. 1818; and London, by Longman and Co.

The use of tartar emetic to excite nausea in favouring the reduction of luxated joints, as proposed by Mr. WILMER, of Coventry,* is next adverted to; though, Dr. Chapman says, he prefers venesection, so as to induce syncope, for this purpose. It should not however be forgotten, that the faintness produced by tartar emetic is but of short duration, whilst that from copious blood-letting is often a thing of no small importance; and this should turn the balance in favour of the former in the greater number of cases.

The following paragraph contains some observations worthy of attention. Dr. Chapman says,

"As an enema, the tartar emetic has proved, in my hands, a most powerful and efficacious remedy, and one which promises hereafter to be of very diversified and extensive application. The first case in which I employed it, was to evacuate the stomach to remove poison which had been swallowed. After having unavailingly tried a series of the most active remedies, I directed that half a drachm of the tartarized antimony dissolved in a little water should be thrown up the rectum; and, as I anticipated, a violent cholera morbus ensued, evacuating the entire alimentary canal, so much so, indeed, that the food undigested came by stool. I have since this time had frequent occasion to resort to the same means, though not under similar circumstances. It has been chiefly in cases of very obstinately obstructed bowels that I have repeated my experiments, and generally with results highly satisfactory. My ordinary prescription for this purpose, is eight or ten grains of the medicine, which I have commonly found sufficient."

The author concludes this article with an account of the substances incompatible with this, in pharmaceutical combination, taken from ORFILA: these are the mineral acids, alkalies and their carbonates, earths, soaps, hydrosulphurets, and the astringent vegetable infusions.

The qualities of tobacco, squills, the preparations of copper, mercury, and zinc, are then passed in review by the author; but he does not here adduce any thing original of importance.

But we cannot follow the author, in the manner we have done in his remarks on emetics, throughout the whole of his work; we must content ourselves with adducing some detached observations, which will give the reader some idea of the nature and value of this system, and recal to his mind some useful observations. The next subject, that of *cathartics*, has received at least sufficient attention amongst us; we shall therefore only remark generally, that it is treated with much judgment by the author, and that his discourse on it is the result of much erudition and judicious reflection.

We think, however, that Dr. Chapman advises the use of cathartics in fever, as we remarked respecting emetics, in too general a manner: the only exception he makes is in yellow-fever, a disease he considers to have the character of a very malignant gastritis. There is nothing in pathology better ascertained than that gastritis is a very common cause of what is often termed idiopathic fever; and therefore, except

* See *London Medical and Physical Journal*, vol. xxiv.

[illegible]n the very first onset of the disease, before the inflammation is established, reasoning would show us that cathartics are as injurious as experience has led Dr. Chapman to believe them to be in yellow-fever. This is too commonly proved by observation. Even at the present day we see purgatives given during inflammation of the mucous membranes of the intestinal canal, to carry off the disordered matter from the bowels; and the practitioner, seeing the quantity of mucus evacuated, felicitates himself on the success of his medicine; goes on inciting this secretion by his poisonous applications to the seat of the disease; and at length laments that malignant, *typhoid*, and putrid, symptoms came on in spite of them, and is surprised that the patient should die, notwithstanding the quantity of wine that he drank, and the bark that was *thrown into* the stomach. Were patients under such circumstances left to follow only the impulse of their instincts,—that is, to abstain from food, and drink cold water,—we should not often witness such results. Thanks to the lights of physiology and the evidence furnished by pathological anatomy, such scenes are now becoming rare.

The impropriety in the precepts to which we alluded, has only arisen from the author having been led, for the sake of conciseness, to speak in too general a manner; for hardly any thing in this work is conceded to blind authority, without reasoning on its propriety; and Dr. Chapman's pathology is not often contrary to that of the most distinguished physicians of the present period, nor his therapeutical measures contrary to the indications it furnishes.

[*To be continued.*]

---

*Reflections on the Nature and Treatment of Cancer, supported by Practical Observations.* By J. J. Lasserre, of Domme, Doctor in Medicine of the Faculty of Paris.*

This memoir contains some observations of remarkable interest to every medical practitioner, whatever general principles of pathology he may have adopted. Even those who consider that *cancer* is, in all cases, essentially a disease of a specific nature, or local morbid action modified by a peculiar diathesis of the system, will not think the cases here related unworthy of their serious attention. We shall therefore select two of these which are the most interesting, and such of the remarks of Dr. Lasserre as are expressly applicable to them; omitting his more general theoretical reflections, because we have already given our readers some ideas of them, on various occasions, in our extracts from the works of M. Broussais, of whose principles the author is a zealous partizan.

"A female peasant, of a dark complexion, robust habit, forty-seven years of age, married since her twenty-third year, and who had never been a mother, experienced, during eight months, great irregu-

---

* *Réflexions sur la Nature et le Traitement du Cancer, avec des Observations à l'appui.* Par J. J. Lasserre, d.m.p. à Domme (Dordogne.) *Journal Universel des Sciences Medicales*, tome xiv.

larities in the periods of the occurrence of the menstrual evacuations, which were always accompanied, too, by violent colics and severe pains about the loins. In the course of the month of May, 1817, she perceived a movable tumor seated above the right nipple, about the size of a large plum. For twelve days she had suffered lancinating pains in the part, which induced her to come to consult me. This was in the ensuing June. The tumor was not painful to the touch; the nipple was redder and more sensible than that of the opposite side. I enquired whether she had ever experienced any external injury to the part, which might have given rise to it, and if she had adopted the bad habit of confining her person tightly by her dress? To these questions she replied in the negative.

"Reduced in this case to regard the affection as consequent on the derangement of the functions of the uterus, and as independant of local mechanic action, I proposed the extirpation of the tumor by the knife.*

"Forced to combat an affection which menaced a rapid progress, from the violence which the pains had acquired for several days past, and the rapid increase of the size of the tumor, I advised the repeated application of leeches to the part itself, and, intermediately, the plaster *de Vigo cum mercurio* mingled with that of cicuta;† an issue in the right arm; half a drachm of mercurial ointment to be rubbed on the same member; bleeding from the arm every eighth day; the use, after intervals of four days, of thirty grains of the pills of Bellost; a severely restricted diet, carried so far as to excite a sensation of *sharp* hunger; and cessation from the use of wine.

"On the 20th of July, thirty-six days from the commencement of the treatment, this woman again visited me. The tumor had diminished to about the size of a small nut; the pains were so slight as to be scarcely sensible; and the nipple was neither redder nor at all more sensible than that of the other side. Menstruation had been totally wanting. The treatment having succeeded beyond my expectations, I ordered its continuance, and added to it the use of whey containing the acetate of potash. Leeches were employed only twice in the ensuing month, as well as the bleedings from the arm.

"The 20th of August, no appearance of menstruation; there did not remain the slightest vestige of the tumor; the pains had ceased for three weeks. The only thing this woman now experienced from her disease, was the emaciation resulting from the forced abstinence, to which she had submitted with a degree of resignation truly exemplary. I have since then frequently seen her: she has regained her health and flesh, and her ordinary gaiety of disposition; not the slightest symptom of her disease has re-appeared. The breast in which the tumor was seated is, however, not so firm and plump as that of the other

* Should we, in symptomatic cancer, have recourse to the operation in the first instance?—*Note of the Editor of the Journ. Univers. des Sciences Méd.*

† "In employing this topic, I had no other object than that of alleviating the pain by means of the sympathy of the skin with the parts which it immediately covers."

side. This woman still preserves the issue open, and wears the plaster on the breast, which she renews every month.

"I should by no means say that I have, on this occasion, cured a cancer of the breast; I am too well convinced that the disease was not a cancerous affection: but I believe I may state that it is highly probable that I have prevented the development of a disease of this nature. Almost all cancers have not a different origin; they are developed under the influence of identical circumstances. If it be demanded of what nature was this woman's disease, I reply, that it was chronic inflammation depending on the influence of sympathy from the uterus; or, rather, from the cessation of the functions of that organ, and the transport to the breast of the sensibility which then abandoned it, perhaps too suddenly.——

—"What I have said respecting chronic engorgement of the glands of the breast, is applicable to disorganizative affections of the neck and body of the uterus. Let us reflect on the circumstances which evidently give rise to cancer of the uterus, and examine them in a physiological point of view, and then see if that malady is not obviously the consequence of prolonged irritation? I need only mention difficult labours, and a want of proper relation between the genital parts of the two sexes in the act of coition. Every physician knows that cancer of the uterus may occur as a consequence of these circumstances.

"This etiology is equally applicable to cancers of the stomach, the rectum, &c. In the greater number of these cases, we only find, if we trace the source of the evil, the slow progress of chronic inflammation, which leads to them as a result. Let it not be said that cancer is a malady *sui generis*, an *essential* malady, an *organic vice*. If all diseases had been regarded in this manner, medicine would have been always left in its infancy. The cultivation of pathological anatomy, the application of the laws of an enlightened physiology, the study of the language of the suffering organs, independantly of the names by which diseases have been designated: such are the only means of making discoveries in medicine really useful to the welfare of patients and to the progress of science. It is to M. Broussais that we owe the first clear exposition of these principles, and the demonstration of the superiority of them by their application to the healing art.

"One case only of chronic engorgement, or, to speak the ordinary language, one case only of schirrus of the uterus, has occurred to me in the course of my practice. Without saying any thing in prejudice of the propriety of the means I employed to combat it, I will here faithfully relate them.

"Mrs. T. forty-three years of age, of a dark complexion, prodigiously fat, and of an ardent disposition of mind, experienced for six months irregularities in menstruation. This lady was the mother of five children: the whole of her labours had been of the most easy and favourable description.

"In the course of the summer of 1817, Mrs. T. began to experience dull pains and a sensation of heat in the interior of the vagina. She complained of it to her husband, who made me acquainted with it, and who much aided my exertions to obtain a full account of the case, by the manner in which he urged her to disclose all the circumstances respecting it. She at length, with extreme difficulty, acknowledged that, for five months past, the approaches of her husband had been the cause of very severe pain. She experienced a sensation of weight in the hypochondres, thrilling pains about the loins and in the groins, and occasionally painful dartings in the lower part of the abdominal cavity, and in the interior of the genital organs. As she was alarmed at these circumstances, (from the recent death of one of her friends, who, after symptoms which she said were similar, had been afflicted with cancer of the uterus,) Mrs. T. permitted me to make an examination of the seat of the disease.

"I found the neck of the uterus hard, unequal, and painful on the slightest pressure: the body of this viscus appeared to be larger than ordinarily, although pressure immediately on it, or above the pubes, did not shew it to be possessed of unusual sensibility. The finger caused a sensation of burning pain in the vagina. The patient had never had any other flux from these parts than the menses and the lochiæ.

"I directed that six leeches should be applied to the vulva, and two days afterwards the same number to the groins, and so on alternately, until they were counter-ordered; a hip-bath daily, made with a strong decoction of poppy-heads; an enema morning and evening, of a slight decoction of the fresh leaves of belladonna; an issue in the left arm; and the most severe restriction in diet, with total abstinence from wine.

"After this treatment had been followed for fifteen days, the pains were much diminished. The patient endured the restriction from food with the greatest impatience; but, on my insisting on the necessity of this measure, she consented to submit to it. Fomentations with the decoction of belladonna were made every evening to the hypogastrium, in addition to the other measures.

"On the 16th of July, the patient had become considerably emaciated; the pains were much less intense. The enemas and fomentation to be discontinued. The leeches were applied only once during the rest of this month. The same severity of regimen. The food consisted solely of vegetables,—as carrots, the fruits of the season, and about four ounces of bread daily. Her drink was merely water and lemonade.

"The 12th of August, the patient suffers no pain, either in the loins, the abdominal cavity, or the groins. The neck of the uterus has regained almost its ordinary size; it is soft, equal, and not painful on pressure; the sensation of burning heat is no longer experienced in the vagina. Menstruation has not appeared since a month before the treatment. The hip-bath to be continued; four grains of extract of cicuta to be taken daily; the abstinence to be less severe.

"The 8th of September, the little tumefaction of the neck of the uterus last noticed has totally disappeared. The treatment was here omitted, and the patient gradually resumed her ordinary regimen. I thought it my duty to represent to her husband the necessity of not again using cohabitation. Since this time no accident has appeared; and I think I may assert that the affected is cured. —— — "In all the cases I have related, it has been seen that I have insisted on abstinence from food to such an extent as to produce *sharp* hunger. The want of alimentation is one of the most favourable conditions for the cure of affections in which it is desirable to effect the re-entry into the circulation of the product of morbid accumulation of nutritive matter, in consequence of inflammation either acute or chronic."

The author concludes with observing, that he has found mercury very efficacious in fulfilling the latter intentions, when employed in the mode, and with the precautions, pointed out by physiology.

---

